# How to Summarize Estimates of Ancestral Divergence Times

**DOI:** 10.4137/ebo.s545

**Published:** 2008-03-18

**Authors:** David A. Morrison

**Affiliations:** Department of Parasitology (SWEPAR), National Veterinary Institute and Swedish University of Agricultural Sciences, Uppsala, Sweden

**Keywords:** lognormal distribution, geometric mean, divergence time

## Abstract

The use of molecular sequence data has increased interest in trying to date evolutionary events, with researchers wanting both an estimate of the divergence time and a confidence interval for that estimate. However, two methodological issues have recently been raised with respect to precision of the estimates: (i) the time of the ancestral event is over-estimated; and (ii) the confidence interval is asymmetrical. I argue that if the estimates of divergence time are considered to be samples from a lognormal probability distribution, then this would explain both of these problems. This implies that divergence times should be presented using geometric means rather than arithmetic means, both for estimates and for their confidence intervals. I present analyses based on both computer simulations and empirical data to show that this approach is effective for both single-gene and multiple-gene data sets. Treating divergence time as a lognormal variable thus provides a simple unifying framework for dealing with many of the problems associated with the estimation of divergence (and possibly coalescence) times. Use of this approach (based on geometric means) can, unfortunately, lead to very different biological conclusions compared to the currently used calculation methods (based on arithmetic means).

## Introduction

The use of molecular sequence data has lead to a renewed interest in trying to date evolutionary events, in addition to simply reconstructing the temporal sequence of those events. Unfortunately, the use of amino acid and DNA data to date evolutionary events is still an uncertain procedure, with several competing strategies of largely unknown reliability. Traditionally, these methods assumed a molecular clock (e.g. distance-based methods) while more recent methods employ various relaxed-clock models (see Magallón, 2004; Renner, 2005; Welch and Bromham, 2005; Rutschmann, 2006). Either way, researchers have been interested in an estimate of the divergence time, often along with a confidence interval for that estimate.

In this regard, two methodological issues have been raised recently with respect to the mathematical calculations, both of which are important but which seem so far to have had little impact on practice: (i) the time of the ancestral event is over-estimated (Nei et al. 2001; Rodríguez-Trelles et al. 2002); and (ii) the confidence interval is asymmetrical (Steel et al. 1996; Haubold and Wiehe, 2001). These have traditionally been treated as separate issues. Here, I point out a possible simple connection between them, and demonstrate that in practice this connection leads to a straightforward and effective procedure for accommodating both of these issues when describing ancestral times.

It is important to note that my concern here is solely with the precision of the time estimates (i.e. their repeatability in response to stochastic variation) rather than with their accuracy (i.e. bias due to non-stochastic variation). A number of issues have been identified that affect the accuracy of the estimates (Magallón and Sanderson, 2005), such as insufficient taxon sampling (Linder et al. 2005), quality and use of fossil calibrations (Rutschmann et al. 2007), small number of loci (Rannala and Yang, 2007), and among-lineage rate variation (Pulquério and Nichols, 2007). Indeed, there are indications that these issues might lead to under-estimation of the divergence times. However, these latter issues of accuracy cannot be addressed effectively until after the issue of precision has been dealt with. Once we know how to present the precision of our estimates we can then proceed to determine their accuracy.

The connection between estimated times and their confidence intervals is not necessarily obvious for methods that produce a single point estimate of an ancestral time (although see the divergence-time histograms of Baldwin and Sanderson, 1998; Kumar and Hedges, 1998; Sanderson and Doyle, 2001; Hedges and Kumar, 2003), but it is immediately obvious for methods (such as those involving markov chain monte carlo, MCMC) that produce estimates based on probability distributions (see, for example, the distributions illustrated by Ballard and Whitlock, 2004; Lemey et al. 2004; Britton, 2005; Huelsenbeck and Ronquist, 2005; Mu et al. 2005). Such distributions are almost always right-skewed (i.e. there is a longer upper tail), as a result of the fact that there is a lower bound to the estimates (i.e. zero time) but no upper bound (at least, not within the time-frame being studied). Here, I argue that if these estimates of divergence time are considered to be samples from a lognormal probability distribution, then this would explain both the over-estimates of times as well as the asymmetry of the confidence intervals. That is, these phenomena are simply two manifestations of the same underlying cause.

The practical upshot of a lognormal distribution is that divergence times should be presented using geometric means rather than arithmetic means, for both estimates and their confidence intervals. This issue seems to be quite general, in the sense that it can affect all of the different methodologies that have been proposed for dating divergence (and perhaps coalescence) events (reviewed by Arbogast et al. 2002; Magallón, 2004; Rutschmann, 2006). Moreover, it is important to note that a lognormal distribution might not be the only possible cause of a right-skewed distribution for divergence times. For example, the curvature of the likelihood function for branch lengths is reduced as the branch lengths get longer, leading to a right skew of the distribution. My contention here, however, is that the lognormal distribution seems to account for a sufficient amount of the skew that it has important practical consequences.

The paper is arranged as follows. I start with a consideration of possible probability distributions for time estimates. I then follow this with first a theoretical and then an empirical assessment of error distributions for single genes, and finally a theoretical and then empirical assessment of error distributions for multiple genes (or loci). I conclude with a consideration of the practical effect of these frequency distributions on biological studies, and some alternative methods for addressing the issues that I have raised.

## Probability Distributions

Rodríguez-Trelles et al. (2002) performed a number of simple computer simulations to show that the frequency histogram of replicate estimates of divergence times is notably right-skewed. Consequently, arithmetic averages of replicate estimates of times will be biased upwards (by up to 35% in their simulations). However, they offered no mathematical insight into this problem, nor did they suggest any possible remedies. In a similar vein, Haubold and Wiehe (2001) noted that confidence intervals on estimated divergence times are usually underestimated, as a result of the fact that the mutation rate for the calibration time is treated as fixed (i.e. known precisely). They provided a mathematical analysis by modelling the variables involved in terms of gamma probability distributions, and produced asymmetrical confidence intervals that reduced the under-estimation in their examples from 45% to 5%.

Mathematically, both of these issues are ultimately related to the probability distribution from which we might expect time estimates to be sampled (i.e. the error distribution). Most of the commonly used divergence methods tacitly assume that the sampling frequency distribution will be a normal (i.e. gaussian) distribution, as this is the conventional assumption in parametric statistical analysis. However, the common point made by Rodríguez-Trelles et al. (2002) and Haubold and Wiehe (2001) is that this assumption is demonstrably incorrect, and that this can lead to substantial over-estimation of the times and under-estimation of the confidence intervals. Unfortunately, there seems to be no analytical proof as to what sampling distribution the relevant time estimates will follow. Indeed, any one of several simplistic models might be equally good as a fit to empirical data.

For example, if taxa are the product of a poisson-based genetic process, as is often assumed in both phylogenetic and genetic analyses, then any measure of divergence between the taxa (i.e. “genetic distance”) will be approximated by a gamma probability distribution. Haubold and Wiehe (2001) essentially used this idea as a basis for deriving a formula to calculate confidence intervals on time estimates, which they showed are more realistic than those derived from assuming a normal distribution.

Alternatively, all of the procedures that assume a molecular clock are based on the idea that the relationship between the “known” calibration time and the unknown time to be evaluated is:

(1) $$\frac{Calibration\;time}{Calibration\;divergence}=\frac{Evaluated\;time}{Evaluated\;divergence}$$

The various methods simply differ in how they measure divergence (e.g. genetic distance, patristic distance, etc.) and how they deal with the associated assumptions/limitations. Three of these terms are to be estimated and are thus associated with more-or-less independent stochastic error; and when calculating the fourth term these stochastic errors are multiplied. Thus, the contributions to the stochastic variation of the final time estimate will be multiplicative, and the estimates will be approximated by a lognormal probability distribution (Galton, 1879). Although they never explicitly noted this fact, Rodríguez-Trelles et al. (2002: 8112) essentially used the idea when they observed that “equivalent random deviations around target times scale divisively forward (i.e. to the present), and multiplicatively backward (i.e. to the past) on their target times.” (Hedges and Kumar (2004) mention the lognormal distribution as a possible error distribution for fossil calibration times, along with the triangular and negative exponential distributions, but they present no explicit rationale for any of these suggestions.)

As a third alternative, in population genetics it is common to invoke a diffusion approximation to the neutral Fisher-Wright model (e.g. Watterson, 1982). Under these circumstances, a brownian-motion model may be appropriate. The inverse-normal (or inverse-gaussian) probability distribution is usually used to model the time that a brownian motion with positive drift takes to reach a fixed positive level, and it has sometimes been used as a model in lifetime studies (e.g. time required to perform some task, length of hospital stays; Seshadri, 1999). This may thus also be a candidate for divergence (or coalescence) times.

So, there are a range of possible distributions that might fit estimates of divergence times in phylogenetic studies, and no obvious theoretical basis on which to distinguish among them. The only available approaches, then, are (i) simulation studies and (ii) the fit to empirical data, both of which I evaluate here.

## Simulated Single-gene Time Estimates

In order to assess the fit of the lognormal (or any other) probability distribution to single-gene time-estimation data, I performed a set of simulations similar to those of Rodríguez-Trelles et al. (2002), which were based on the assumption of a molecular clock. This is the simplest form of analysis for divergence times, so that it is a subset of all other analyses (e.g. those based on relaxed-clock models). This implies that the conclusions are likely to apply to the other analyses as well.

My simulations used a 3-taxon rooted tree, with the calibration time specified for the ancestral node of the closest two taxa and the time to be estimated as the root node. The target time expected in the simulations was set to 2, 5 or 10 times the age of the calibration node. The branch lengths of the tree were then systematically varied (1, 2, 5, 10, 20, 30, 40, 50, 60, 70, 80, 90, 100), thus emulating either varying evolutionary time (if the substitution rate is considered to be fixed) or varying substitution rate (if the evolutionary time is considered to be fixed), in combination with systematic variation of the amino-acid sequence lengths (100, 150, 300, 600). Sequences were simulated along the tree using the Jones-Taylor-Thornton substitution model, with α = 1 for the among-site rate variation, using the PSeq-Gen ver. 1.1 program (Grassly et al. 1997). There were 1000 replicate simulations for each of the 156 data sets (i.e. all combinations of 3 calibration times, 13 branch lengths and 4 sequence lengths). Simulations have indicated that sample sizes >500 typically give strong support to a single model when assessing the fit of data to a probability distribution (Dick, 2004), and so 1000 replicates should be ample to assess whether a candidate distribution is a better fit than any of the alternatives.

The genetic distances among the sequences in each data set were then estimated using the ProtDist program of the Phylip ver. 3.6a3 package (Felsenstein, 2002), specifying the same substitution model parameters as for the simulations (i.e. a best-case scenario for evolutionary reconstruction under a molecular clock). This procedure may, however, produce slight additional variation due to the differences in the way the Jones-Taylor-Thornton model is implemented in PSeq-Gen compared to Protdist (Kosiol and Goldman, 2005). The time estimates were then calculated by dividing the average genetic distance between the estimation sequence and the calibration sequences by the genetic distance between the two calibration sequences.

The frequency distribution of the 1000 replicate times for each data set was then compared to a normal probability distribution and a lognormal distribution via maximum-likelihood fitting, using the Regress+ ver. 2.3.1 program (McLaughlin, 1999). This program was also used for all of the other maximum-likelihood fitting of frequency distributions to data, as discussed below. Since the gamma probability distribution can also be used as a model for multiplicative errors (Firth, 1988), and Thorne and Kishino (2002) model the age of a cladogram root using a gamma prior, a 2-parameter gamma distribution was used as a comparison for the fit of the lognormal to the data. I also tested the fit of both the inverse-normal and the 2-parameter weibull distributions as potential approximations to the time-estimate data. The latter has found empirical favour as a fit to right-skewed frequency histograms in other areas of biology, most notably in studies of survival time (Collett, 2003; Lee and Wang, 2003). This is an extreme value distribution, used when dealing with the maximum values of a series of observations that have an upper limit, and is thus a generalization of the gamma distribution to situations where there is not a constant event-probability through time.

For almost all of the data sets examined, the rank order of the log-likelihood values was the same: lognormal > gamma > normal. The only exceptions to this were the five data sets with the largest log-likelihood values for the normal distribution, in which case the gamma distribution was a slightly worse fit to the data than was the normal. However, the lognormal probability distribution always provided a better fit to the data than did either the gamma or the normal distributions, even when the likelihood values were very close. Unfortunately, the lognormal distribution was sometimes detectably different from the data distribution, as determined by Kolmogorov-Smirnov goodness-of-fit tests (using Regress+), especially for short sequence lengths and/or short branch lengths. Thus, the data do not flawlessly fit a lognormal distribution, although this is a better approximation than is either of the alternative error distributions tested.

The weibull probability distribution was never a good fit to the data, and in fact was only a better fit than the normal distribution for less than one-third of the data sets. The inverse-normal probability distribution, on the other hand, was usually just as good a fit to the data sets as was the lognormal distribution, although the lognormal had the edge in two-thirds of the cases. This is not necessarily surprising, as the inverse-normal and lognormal probability distributions are very similar if the coefficient of variation (CV; see below) is <1 (Takagi et al. 1997). Thus, there is no clear-cut evidence that the lognormal distribution is *always* the true generating distribution of divergence times, although it seems to be the most likely of the candidates tested.

So, while I can make no strong mathematical claim that evolutionary time estimates *are* sampled from a lognormal distribution, even for data that fit the molecular clock, I suggest that it is the distribution for which the most effective case can be made. Next, I explore some of the practical consequences that follow if this suggestion is true, noting that there are several important advantages.

## Presenting Divergence Estimates

If a frequency distribution is lognormal, then the geometric mean will be a more natural measure of the central location of that distribution than will the arithmetic mean. That is, if divergence time is a lognormal variable then it will show multiplicative stochastic variation around the geometric mean (rather than additive variation around the arithmetic mean, as for a normal variable); and the geometric mean will always be ≤ the arithmetic mean (Muirhead, 1903). Furthermore, the confidence intervals of a lognormal variable are asymmetrical, and are larger than are those of a normal distribution. These combined characteristics clearly have the potential to address both of the problems that I identified above for the currently used divergence-time procedures.

The geometric mean is preferred to the arithmetic mean (in this context, but not necessarily in other contexts; e.g. Parkhurst, 1998) for a number of inter-related reasons (Limpert et al. 2001), and this is what makes the lognormal distribution (and the associated logarithm transformation) unique among non-normal probability distributions (Keene, 1995). The geometric mean is the back-transformed value of the arithmetic mean of the log-transformed data. For a lognormal distribution, this version of the mean will thus possess many of the desirable properties associated with the arithmetic mean of a normal distribution, which are *not* possessed by the arithmetic mean of the lognormal distribution itself (Schmoyer et al. 1996). This follows from the fact that a lognormal distribution is normally distributed on the logarithmic scale. These properties include the fact that the sample mean is not an efficient estimator of the population arithmetic mean (Mehran, 1973) and that the geometric mean will equal the median (or be an efficient estimator of the median for small samples sizes). Furthermore, the geometric mean is zero if *any* of the component observations is zero.

**Figure 1:**
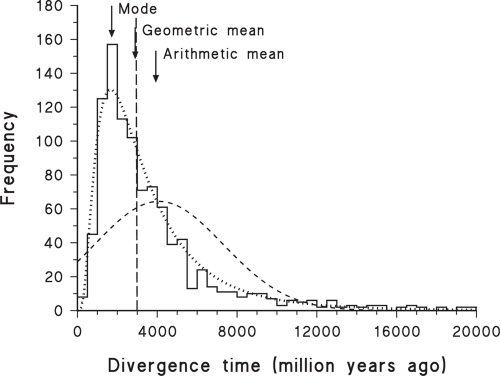
Frequency histogram of 1000 simulations of a single estimated divergence time. The simulations were based on a 3-taxon rooted tree, with the calibration time specified for the ancestral node of the closest two taxa and the time to be estimated as the root node. The target time expected in the simulations is shown as the vertical long-dashed line, along with the best-fit normal (dashed line) and lognormal (dotted line) probability distributions. The fit of the data to the lognormal probability distribution is good over most of the range, but the second and fourth histogram bins are significantly different from their expectations, as determined by a goodness-of-fit test (G = 40.20, p = 0.020). The original data are from Rodríguez-Trelles et al. (2002).

A practical demonstration of these theoretical properties is illustrated in Figure 1, which shows the results of one of the computer simulations of Rodríguez-Trelles et al. (2002). The target time to be estimated in the simulations was 3,000 million years, and 1,000 replicate simulations were performed. As can be seen, the data are a reasonable fit to the lognormal distribution (as estimated by maximum likelihood), and therefore the geometric mean is a good estimator of the target time, while the arithmetic mean provides a large over-estimate. This will always be true for data that fit a lognormal probability distribution, and will be approximately true for data that approximate a lognormal.

Confidence intervals for a lognormal variable are easily calculated by estimating the confidence limits on the logarithmic scale and then back-transforming them to the original scale. This will produce asymmetrical confidence intervals, which will extend further towards larger values than will the equivalent confidence intervals based on the normal distribution.

A practical demonstration of this point is illustrated in Table 1, which shows the results of the empirical data analysed by Haubold and Wiehe (2001). The empirical confidence intervals were estimated using bootstrapping, and then these were approximated by the gamma-based method devised by Haubold and Wiehe (2001). The confidence intervals for the same data derived by assuming that the data fit a lognormal distribution are also shown. As can be seen, in all cases the lognormal-based confidence intervals are a reasonably good fit to the “true” values, and are as good an approximation to these values as are the gamma-based confidence intervals. This is to be expected if the data are a good fit to a lognormal frequency distribution. Moreover, the lognormal-based confidence intervals are simpler to calculate than are the gamma-based confidence intervals.

It is important to note that many researchers prefer to quote the mean (*x*) and standard deviation/error (*s*) rather than confidence intervals (Hedges and Kumar, 2004), a practice that Graur and Martin (2004) rightly criticize in the context of estimating evolutionary times, since it can give a false impression of precision to those readers who do not mentally turn the values into confidence intervals. Moreover, this practice is clearly problematic if the data are non-normal, because the usual convention of reporting *x* ± *s* will not work, since the confidence intervals are not symmetrical about the mean. (Hedges and Kumar (2004: 245) note that time estimates have a skewed distribution but still claim that use of standard errors “is a matter of choice”, apparently not noticing the logical contradiction.) Limpert et al. (2001) remark that an alternative convention for lognormally distributed data is to report *x*′ ^x^/*s*′, where *x*′ = exp(*x*), *s*′ = exp(*s*) and *x* and *s* are calculated on the log-transformed scale (i.e. *x*′ is the geometric mean). Here, ^x^/stands for “times divide” (by analogy with “plus minus” for±), so that multiplying *x*′ by *s*′ has the same interpretation as adding *s* to *x*, and dividing *x*′ by *s*′ has the same interpretation as subtracting *s* from *x*. However, quoting the confidence intervals directly is clearly a much simpler convention for all concerned.

The geometric mean can be calculated straightforwardly from the arithmetic mean (*Mean*) and standard deviation (*StDev*):

(2) $$\begin{array}{l} Geometric\;Mean\\ =\exp\left(\left(\ln(Mean\right)-\frac{\ln\left[{\left(\frac{StDev}{Mean}\right)}^{2}+1\right]}{2}\right) \end{array}$$

So, any time-estimate method that produces an arithmetic mean and standard deviation (or a single estimate and its standard error) can be used to calculate the geometric mean and its associated confidence interval—and all estimates involve a standard error, even if the latter is neither calculated (by the program) nor reported (by the researcher). However, this formula is not particularly efficient for large standard deviations (Gaddum, 1945), and so it is always better to use the raw data for calculations. Note, incidentally, that the r8s computer program (Sanderson, 2006) produces a point estimate of the divergence time but the current version does not provide any information that could be used to calculate a confidence interval (or the geometric mean); instead, a complicated bootstrapping procedure is recommended.

**Figure 2:**
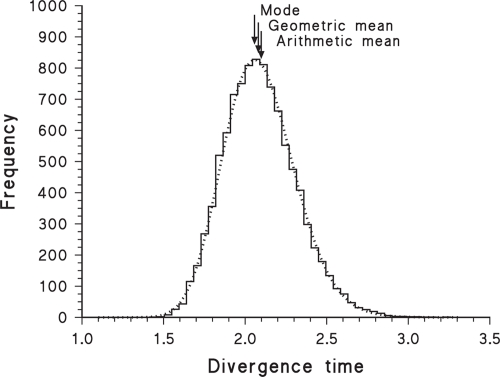
Frequency histogram of 100,000 bayesian samples of the estimated divergence time of four taxa, based on a single locus, as analysed by the Multidivtime computer program. The time scale is relative (i.e. unitless until a substitution rate is specified for each locus). Also shown is the best-fit lognormal probability distribution (dotted line). The original data are from Gene 3 of the example data set distributed with the Multidivtime computer program.

The geometric mean can also be used for methods that produce an empirical probability distribution rather than a point estimate of the divergence time (e.g. many of the relaxed-clock methods, as listed by Rutschmann, 2006). That is, when confronted with the output from a computer program that uses, for example, MCMC methods to produce a frequency distribution of estimated divergence times, it is not necessarily obvious whether to use the mean, median or mode as the preferred point estimate. I am suggesting using the geometric mean, which unfortunately is not a number calculated by any of the currently available programs, all of which seem to use either the arithmetic mean or the mode (e.g. Beast: Drummond and Rambaut, 2007; IM: Hey and Nielsen, 2004; Multidivtime: Thorne and Kishino, 2002; PAML: Yang, 2007; Path: Britton, 2005; PhyBayes: Aris-Brosou and Yang, 2002; Qdate: Rambaut and Bromham, 1998; r8s: Sanderson, 2006; Timer: Glazko and Nei, 2003). For example, Figure 2 shows the output from analysis of a single gene by the Multidivtime ver. 09.25.03 program (Thorne and Kishino, 2002), illustrating the good fit of the bayesian posterior frequency distribution to a log-normal probability distribution (lognormal log-likelihood = 1017.02; normal log-likelihood = 871.63), for which the geometric mean would then be appropriate.

Note, incidentally, that a bayesian credible interval is not the same thing as a confidence interval on a point estimate. A bayesian analysis does not produce point estimates of a parameter (such as a mean), but instead considers the whole sample as being the appropriate solution to the analysis (since it deals with random variables rather than statistical estimates of parameters). Thus, a credible interval refers to the whole sample, rather than to any point estimate derived from that sample (such as a mode, median or mean). A credible interval is thus likely to be a poor estimate of the confidence interval of the geometric mean. (This fact does not deny the potential usefulness of a credible interval in its own right.)

**Figure 3:**
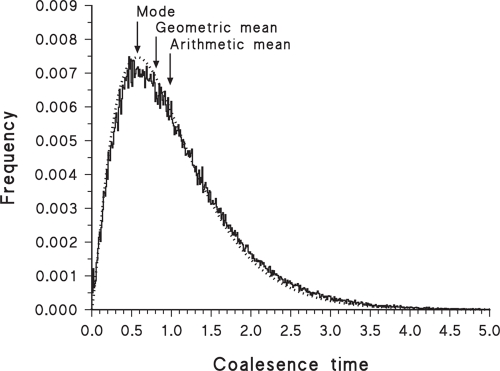
Frequency histogram of 300,000 bayesian samples of the estimated coalescence time of two populations, based on a single locus, as analysed by the IM computer program. The time scale is relative (i.e. unitless until a substitution rate is specified for the locus). Also shown is the best-fit gamma probability distribution (dotted line). The original data are from Morrison (2005), based on α-tubulin intron sequences of lineages I and II of *Toxoplasma gondii* (Apicomplexa).

It is also important to note that it is possible to find analyses of data sets that fit a gamma probability distribution better than they do a lognormal distribution. For example, the IM program of Hey and Nielsen (2004) uses a MCMC procedure to estimate demographic parameters under a specified model of divergence for a pair of populations from their common ancestral population. When estimating coalescence (rather than divergence) time based on a single locus, this program often produces bayesian posterior frequency histograms, as shown by the example in Figure 3, where the gamma distribution is a much better fit to the data (log-likelihood = −856.67) than is either the log-normal (log-likelihood = −933.80) or the normal (log-likelihood = −954.05) probability distributions. This program incorporates the coalescent into the ancestral population, which involves a convolution of exponential distributions—a gamma distribution is thus not an unexpected result for the posterior probability distribution. Similarly, the simulated coalescence distribution shown by Ballard and Whitlock (2004) (their Fig. 2) fits a gamma distribution better than a lognormal distribution. This does not preclude use of the geometric mean and its confidence interval for such analyses, but it does emphasize that the rationale for doing so is only a convenient approximation in these cases.

Finally, it is perhaps worthwhile to point out that the sum (or difference) of two lognormal variables is not lognormally distributed (Blackwood, 1992). Indeed, the resulting probability distribution has a distinctly awkward mathematical form (Naus, 1969). This will presumably make it difficult to calculate confidence intervals for estimates of differences between divergence times. Furthermore, statistical tests of geometric means involve their ratios rather than their differences (as is the case for arithmetic means), because the tests are performed on the logarithm-transformed data (Blackwood, 1992). Rejecting a null hypothesis for equality of geometric means thus implies more than just a shift in the central tendency of the untransformed data, as the mean and standard deviation are confounded on the original scale (see equation 2).

Incidentally, the confounding of the mean and standard deviation leads to the result that for a lognormal variable the width of the confidence interval will be directly related to the size of the mean, since the confidence interval is calculated from the standard deviation. Thus, the fact that Yang and Rannala (2006) and Rannala and Yang (2007) both observed a linear relationship between these variables in their bayesian analyses implies that their simulated data also follow lognormal distributions. The latter conclude that “the slope of the regression line [between the size of an arithmetic mean and its confidence interval] indicates the amount of uncertainty in posterior time estimates that cannot be removed by increasing sequence data” (Rannala and Yang, 2007: 462)—this characteristic is another inevitable consequence of divergence time being a lognormal variable.

## Empirical Single-gene Time Estimates

This brings us to the practical issue of how much effect using the geometric mean rather than the arithmetic mean is likely to have in practice. I will consider time estimates based on single genes first.

As shown by equation (2), for a lognormal variable the standard deviation is not independent of the arithmetic mean. One consequence of this is that the lognormal frequency distribution approaches the normal frequency distribution as the standard deviation decreases (Evans et al. 1993). This implies that the value of the geometric mean approaches that of the arithmetic mean as the standard deviation decreases. Consequently, the two problems being discussed here are reduced for any time-estimation method or data set that has a smaller standard deviation—the smaller the standard deviation then the smaller will be the difference between the arithmetic and geometric means and the smaller and more symmetrical will be the confidence interval.

**Figure 4:**
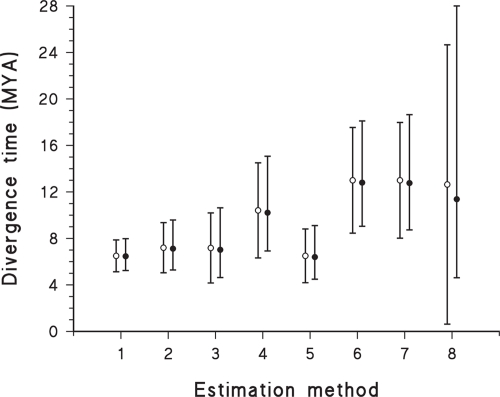
Divergence time estimates (million years ago) for the gorilla—human split (using the human—orangutan split as the calibration time) based on eight different estimation methods, which produce different coefficients of variation (CV), and using the arithmetic (open symbols) and geometric (filled symbols) means and 95% confidence intervals. The methods are 1–2: protein gamma distance (Glazko and Nei, 2003), with 1 = concatenated sequences, 2 = individual sequences; 3–4: bayesian (Aris-Brosou and Yang, 2002), 3 = molecular clock, 4 = non-clock; and 5–8: maximum likelihood (Aris-Brosou and Yang, 2002), 5 = molecular clock, 6 = two rates, 7 = three rates, 8 = four rates. For method 1 CV ≈ 10%, for methods 2, 5 CV ≈ 15%, for methods 3, 4, 6, 7 CV ≈ 20%, and for method 8 CV ≈ 50%.

This is illustrated for a specific empirical example in Figure 4, which shows a range of estimates for the same divergence time based on the same calibration time, but using different estimation methods. For the method with the smallest coefficient of variation (the standard deviation as a percentage of the mean) the arithmetic and geometric estimates are almost identical, whereas the method with the largest coefficient of variation (CV) has distinctly different arithmetic and geometric means and confidence intervals. In this case, the difference is not trivial because the 95% confidence intervals for the geometric means indicate that the estimates based on methods 6 and 7 are not the same as the estimate from method 5 (the latter assumes a molecular clock while the former do not) while in contrast the confidence intervals for the arithmetic means do overlap.

It is also worth pointing out that if the standard deviation is larger than 50% of the arithmetic mean then the data cannot be normally distributed, as the 95% confidence interval would then include negative numbers (i.e. for a normal probability distribution, 95% CI ≈ estimate ± 2 × standard deviation of estimate), which is illogical for divergence times. This is a simple heuristic test of whether or not the assumption of a normal distribution is valid. This point is illustrated for method 8 in Figure 4, where the confidence interval of the arithmetic mean suggests that we can have no confidence at all in the divergence time (i.e. it could almost include zero) while the lower bound for the confidence interval of the geometric mean is quite in accord with the (non-zero) lower bound for the other estimation methods. The geometric mean and its confidence interval will always be non-negative.

Since the degree of non-normality is related to the magnitude of the standard deviation, all circumstances that reduce the standard deviation of a single-gene estimate will reduce the effect. These can include: (1) increasing the sequence length; (2) increasing the evolutionary branch lengths relating the taxa, either by increasing the substitution rate or by increasing the evolutionary time, so that the number of inferred substitutions is increased (although saturation will then eventually become a problem); (3) decreasing the time difference between the estimation point and the calibration point; and (4) use of an adequate evolutionary model for estimating sequence divergence. Rodríguez-Trelles et al. (2002) provide the results of computer simulations to demonstrate some of these points, and Nei et al. (2001) provide a mathematical analysis to demonstrate some of the others.

As far as empirical data are concerned, most of the suitable data sets that I have investigated (i.e. those few that provide sufficient information to examine the shape of the sampling distribution) show a better fit to a lognormal than to a normal distribution, thus confirming the simulation study. However, approximately normal distributions can certainly be found, as shown for example by the data of Baldwin and Sanderson (1998). These are based on ITS sequences of the Hawaiian silversword alliance (Asteraceae), using 100 bootstrap replicates of an estimated time to the most recent common ancestor (TMRCA), illustrating stochastic variation due to character sampling of a single gene sequence. Here, the normal distribution is almost as good a fit to the data (log-likelihood= −120.43) as is the lognormal (log-likelihood = −118.87). In such cases, use of either the geometric or arithmetic mean will have little effect on the time estimates and their confidence intervals.

Also, different sources of stochastic variation may have different effects on the frequency distribution. Table 2 shows one example of this phenomenon. Three different sources of stochastic variation were examined for this data set, only one of which (Topological uncertainty) shows a better fit to a lognormal probability distribution than to a normal distribution. This source of variation produces distinctly skewed sampling distributions, while the other two sources produce more symmetrical distributions. Indeed, for the other two sources of variation, situations were encountered where the distribution was slightly negatively skewed, rather than positively skewed, under which circumstances the lognormal cannot be a better fit to the data than is the normal.

The possible magnitude of the effect of using the geometric rather than the arithmetic mean can be illustrated by a comparison of two empirical data sets based on the same gene sequence (plant *rbc*L) but estimating events at different times and using estimation methods with different standard deviations. The first data set is from Xiang et al. (2000), on the timing of the Asian—American disjunction for selected pairs of related species, with time estimation via the number of synonymous substitutions. These data have relatively large standard deviations for each of the 10 estimates (34%–71% of the mean), and so the inferred lognormal distribution would be distinctly non-normal. Thus, the geometric mean is very different from the arithmetic mean for these data (5%–19% smaller). The second data set is from Britton et al. (2002), on the timing of all of the nodes on a single phylogenetic tree for selected members of the Liliales, with time estimation via the mean pathlengths on the tree. These data generally have much smaller standard deviations for each of the 39 estimates (6%–41% of the mean) and thus there is much less difference between the inferred lognormal distribution and the normal one, so that the geometric mean is not much different from the arithmetic mean (1%–7% smaller).

These two data sets are typical of those that I have encountered. From this, I infer that the effect of using the arithmetic mean instead of the geometric mean is not likely to lead to major overestimation of divergence times under realistic circumstances, as most studies use sufficient sequence data to keep the standard deviation to a relatively small percentage of the mean. Nevertheless, unnecessary (avoidable) over-estimations of up to 20% are possible when using the arithmetic mean compared to use of the geometric mean.

## Time Estimates Based on Multiple Genes

The most common method for estimating nodal times based on multiple gene (or locus) sequences has been to average the estimates obtained for each gene individually. It is, however, possible to concatenate the gene sequences and to obtain a single estimate, which would in principle be the same as using the single-gene methods discussed above. Alternatively, there are recently developed methods that explicitly provide both individual and combined estimates for multiple genes, including the bayesian methods of Hey and Nielsen (2004) for population genetics, and those of Thorne and Kishino (2002), Drummond et al. (2006) and Rannala and Yang (2007) for phylogenetics, as well as the multiprotein gamma distance method of Nei et al. (2001).

If the stochastic variation between genes produces multiplicative errors, then it can be expected that estimates of a single time averaged across multiple genes will also approximately follow a lognormal probability distribution, using the same argument presented above, irrespective of whether they are arithmetic or geometric estimates (Evans et al. 1993). However, the Central Limit theorem indicates that the distribution of the arithmetic means will approach a normal distribution as the sample size increases (i.e. the number of genes), since arithmetic means are normally distributed in the limit, while the distribution of the geometric means will remain lognormal (Blackwood, 1992). What is unknown (and probably unknowable) is how fast these limits will be approached with increasing amounts of data. (Note that Rodríguez-Trelles et al. (2002: 8114) incorrectly suggest that “averages across multiple measures of the same divergence time are expected to converge to more consistent over-estimates as molecular data sets become vastly larger in the future.”)

If the geometric mean estimates for individual genes are closer to the true value than are the arithmetic estimates, then the standard deviation of the overall average will be smaller, and it is thus reasonable to expect that geometric means will be approximately normally distributed. Consequently, it may not matter much in practice whether one uses arithmetic or geometric averages across genes provided that geometric means have been used for each gene. However, even if arithmetic means are used for each gene, a geometric average of these means should still be closer to the true time value, if the overall distribution is approximately lognormal.

As a heuristic assessment of these two predictions, I performed a simulation experiment for a single time estimation based on 20 replicate genes. Each gene was simulated on a 3-taxon tree, as described above (i.e. 1000 simulated data sets per gene, target time set to 10 times the calibration time, Jones-Taylor-Thornton substitution model), but the characteristics of each gene were sampled at random from the following: (a) the sequence length was chosen from a uniform distribution with range either 150–300 or 300–600 amino acids (with equal probability for the two ranges); (b) the gamma parameter for the among-site rate variation was chosen from a lognormal distribution with mean of 0.60 and standard deviation of 1.00 (on the log scale; mean = 1.8 on the normal scale); and (c) the branch length was chosen from a uniform distribution on a log_10_ scale with range 0–2 (i.e. 1–100 on the normal scale). The latter characteristic simulates among-gene variation in substitution rate in this case, since the calibration time is assumed to be the same for all of the genes. These appear to be realistic conditions for protein-coding sequences, based on the published literature, thus representing phoney real data (or perhaps genuine phoney data).

The unknown divergence time was estimated in all of the four possible combinations: (i) the arithmetic average across genes of the arithmetic mean for each gene; (ii) the geometric average across genes of the arithmetic mean for each gene; (iii) the arithmetic average across genes of the geometric mean for each gene; and (iv) the geometric average across genes of the geometric mean for each gene. The use of the geometric mean for each gene produced estimates that varied little across the genes (Table 3), so that the form of averaging procedure across the genes had little effect, with overall only a 3% over-estimate of the target time (i.e. 10 time units) using either method. However, use of the arithmetic mean followed by arithmetic averaging (the current standard procedure) produced an over-estimate of 15%, which was reduced to 12% by using the geometric average across genes (Table 3). This occurred because the arithmetic means were a better fit to a lognormal distribution than to a normal distribution (as estimated by likelihood fitting, Table 3), producing a large spread of the estimates (as measured by the standard deviation, Table 3). Thus, both of the above predictions are confirmed for this simple example.

However, Nei et al. (2001) provide an alternative mathematical analysis of the multi-gene estimation of divergence times. They point out that averaging across single-gene estimates is not an unbiased procedure, as the final time estimate will have extra components related to the variances and covariances of the estimates within each gene (their Equation 3). It is these extra components that lead to the over-estimation, in a multiplicative fashion. They present an unbiased distance-based procedure for estimating the time for each gene, which is also shown in Table 3 for the simulated data. Clearly, this estimator leads to an almost perfect normal distribution across the genes, as expected from the Central Limit theorem, and a good estimation of the true divergence time. However, the use of the geometric mean for each gene leads to a good approximation to this unbiased estimator, thus demonstrating that a single consistent viewpoint (i.e. that divergence time is a lognormal variable) does effectively unite many of the existing methods into a coherent framework.

There are also methods for estimating coalescence times from multiple genes that simultaneously estimate the ancestral population size as well (see Arbogast et al. 2002; Wall, 2003). I have not directly addressed these methods here.

## Empirical Multi-gene Time Estimates

A survey of the small amount of available literature indicates that time estimates from multi-locus data sets may or may not show a better fit to a lognormal probability distribution than to a normal distribution (see also Hedges and Kumar, 2003). This is illustrated by the example in Table 4, which shows six multi-gene time estimates based on arithmetic estimates for each gene. Three of the six time estimates have strongly skewed frequency distributions for these arithmetic estimates, and these thus fit the lognormal distribution much better than they fit the normal distribution. For all three of these cases, there is a notable difference between the geometric average of the single-gene estimates and the arithmetic average, comparable in magnitude to the effect shown above for the single-gene data sets (i.e. up to 15% difference).

It is probable that this effect size is general for multi-gene estimates, as illustrated by two further examples. The first example is from Heckman et al. (2001), on the timing of divergence for selected pairs of higher taxa of fungi and plants, based on averaging of single-gene arithmetic estimates. It shows that the geometric mean can be quite different from the arithmetic mean (1%–14% smaller for each of the 10 estimates), as the inferred lognormal distribution would be distinctly non-normal in many of the cases. The second example is from Hedges et al. (2004), on the timing of all of the nodes on a single phylogenetic tree for selected higher taxa of eukaryotes, based on first concatenating the genes and then calculating a single time estimate (and this study also used a larger data set). This example shows much less difference between the inferred lognormal distribution and the normal one, so that the geometric mean is not much different from the arithmetic mean (<2% smaller for each of the 17 estimates). This similarity may be a general property of concatenating the genes.

Note, however, that only two of the three non-skewed frequency distributions shown in Table 4 have similar geometric and arithmetic means (e.g. <10% difference), while the Fungi—Plant divergence estimate still shows a disparity between the two. This occurs because there are two outlying small values in this part of the data set, and the calculation of the geometric mean is sensitive to these values (i.e. they considerably reduce the value of the geometric mean). If these two values are excluded, then the arithmetic mean = 1742 MYA, the geometric mean = 1607 MYA, and their difference = −7.7%; this pattern is now in accord with those of the other two divergence times. This example thus shows that using the geometric mean of multi-gene estimates cannot be done indiscriminately—this calculation has its own assumptions that must be met. It will always be better to calculate geometric estimates for each gene and then to average these, thus correcting for the over-estimation problem at the source, rather than to calculate arithmetic estimates for each gene and then trying to average these in some less-appropriate way.

**Figure 5:**
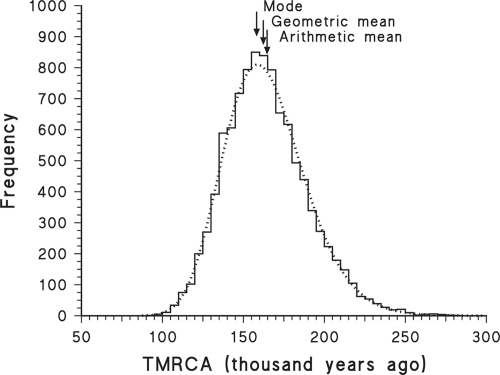
Frequency histogram of 100,000 bayesian samples of the estimated coalescence time of six lineages, based on seven concatenated loci, as analysed by the Beast computer program. Also shown is the best-fit lognormal probability distribution (dotted line). The original data are from Morrison (2005), based on seven house-keeping gene sequences of lineages I, II and III plus samples Castells, Cougar and MAS of *Toxoplasma gondii* (Apicomplexa).

Those methods that produce an empirical probability distribution rather than a point estimate of the divergence time may or may not produce something that is closer to a lognormal distribution than to a normal distribution. For example, analysis of the example 3-gene data set provided with the Multidivtime ver. 09.25.03 program (one gene of which is shown in Fig. 2) produces a posterior distribution that fits a lognormal only slightly better than a normal distribution. On the other hand, Figure 5 shows an example of the analysis of a concatenated 7-gene data set by the Beast ver. 1.2 program (Drummond and Rambaut, 2007). The bayesian posterior frequency distribution fits a lognormal probability distribution very well (lognormal log-likelihood = −46349.3; normal log-likelihood = −46585.5).

**Figure 6:**
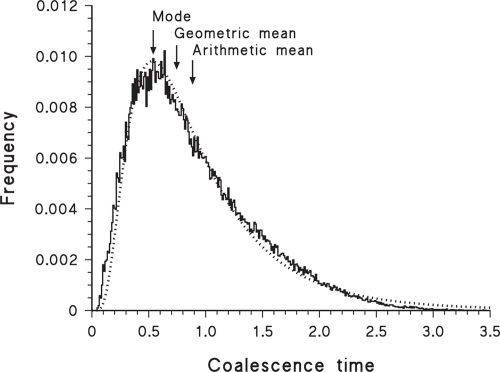
Frequency histogram of 300,000 bayesian samples of the estimated coalescence time of two populations, based on seven loci, as analysed by the IM computer program. The time scale is relative (i.e. unitless until a substitution rate is specified for each locus). Also shown is the best-fit lognormal probability distribution (dotted line). The original data are from Morrison (2005), based on seven antigen-coding gene sequences of lineages I and II of *Toxoplasma gondii* (Apicomplexa).

Finally, it is instructive to return to the IM program of Hey and Nielsen (2004), which it was pointed out above can produce distinctly gamma-like frequency histograms for single genes. For multi-locus data sets, I have observed that this program is more likely to produce lognormal frequency distributions, as illustrated by the example in Figure 6. The program models variation in substitution rates across the loci in a multiplicative manner, and so this result is not surprising (i.e. the multiplicative effects outweigh the other stochastic effects).

## Does the Estimation Method Matter for Time Estimates?

This inevitably leads to the question of whether the difference between arithmetic and geometric means is large enough to produce contradictory conclusions. Unfortunately, it is easy to find examples where this is so.

As a first example, consider the empirical data set shown in Table 5. This involved analysis of 29 amino acid sequences for the primate—rodent divergence time (expected to be 110 MYA), using the primate—artiodactyl divergence (90 MYA) as the calibration time. I measured divergence as the poisson-corrected gamma distance and its variance as described by Nei et al. (2001), using the Timer ver. 0.1 program of Glazko and Nei (2003). Thus, I obtained arithmetic divergence estimates and standard errors for each gene, and these were converted to geometric estimates using equation (2); and subsequently I calculated both arithmetic and geometric averages across the genes. The divergence time was thus estimated in the four different ways described for the multi-gene simulation above. Use of the geometric average of the geometric estimates produced a time estimate that is in close accord with the estimate produced by the multiprotein gamma-distance method of Nei et al. (2001), albeit with a larger confidence interval, and which is also closest to the expected value (although that value itself may not be correct, of course). Calculation of the arithmetic average of the arithmetic estimates (i.e. the current standard procedure) leads to a confidence interval that does not include the expected value (Table 5), which means that this procedure would result in a different conclusion about whether or not that value (110 MYA) is supported by the data, since use of the geometric average in any way leads to a confidence interval that includes the expected value. Thus, using the confidence intervals as hypothesis tests leads to contradictory conclusions for the arithmetic versus geometric means (irrespective of whether the 110 MYA is the true value or not).

As a second example, Shaul and Graur (2002) illustrate their (justified) concerns about reciprocal consistency of calibration points by re-analysing the multi-gene data of Wang et al. (1999). These authors produced estimates for the primat—rodent divergence time based on the bird—mammal divergence time as a calibration, and vice versa. I calculated the sequence divergence between the 29 pairs of taxa as the poisson-corrected distance and its variance as described by Nei et al. (2001), using the MEGA ver. 2.1 program of Kumar et al. (2001). The two divergence times based on the data for each gene were then calculated using the formulae presented by Shaul and Graur (2002). The standard error of each time estimate was calculated by combining the errors for the component genetic distances, using standard methods based on quadrature (Taylor, 1997). These standard errors were then used to calculate the geometric mean and confidence interval for each estimate. On this basis, the bird—mammal divergence (T2) is estimated to be statistically significantly earlier than the primate—rodent divergence (T1) for 14 of the 29 gene sequences (Table 6), based on non-overlapping confidence intervals, and in no case is T1 > T2 (i.e. in all other cases the two confidence intervals overlap). This conclusion contrasts with that of Shaul and Graur (2002) based on arithmetic point estimates (i.e. arithmetic means without confidence intervals), who decided that for 7 of the 29 genes T1 > T2, thus leading them to call into serious question the use of secondary calibration points. So, while Shaul and Graur’s concern about the weaknesses of secondary calibration points may be valid, the data of Wang et al. (1999) do not provide a suitable example of any such weakness.

This example is an important one, because the conclusions of the paper by Shaul and Graur continue to be cited, even though there appears to be no empirical evidence for these conclusions.

## Alternative Strategies for Multiple Genes

I have argued here that many of the estimation problems of divergence times are simply a product of the fact that the times are being sampled from a lognormal rather than a normal distribution (i.e. divergence time is a lognormal variable). I thus claim that this approach provides a unifying framework for dealing with many of these problems. There are, however, possible alternative viewpoints of the situation.

One alternative view of the skewed frequency distribution of time estimates for multiple genes is that it represents a symmetrical distribution contaminated by outliers (Hedges and Kumar, 2003), possibly arising as the result of a small sample size (Hedges and Kumar, 2004). From this viewpoint, the correct procedure would be to deal with the bias caused by the outliers in some appropriate manner, by using what are known as robust statistical procedures. Strategies that have been used for divergence-time estimation include: excluding those outliers explicitly detected by a statistical test (Shaul and Graur, 2002); using a 10% trimmed mean (Kumar and Hedges, 1998; Wang et al. 1999); using the mode (Heckman et al. 2001; Hedges et al. 2004; Hedges and Kumar, 2004); and using the median (Feng et al. 1997; Aris-Brosou and Yang, 2002; Hedges and Kumar, 2004).

These alternative strategies all have problems (see below), relative to the more straightforward use of the geometric mean that I am advocating. They are procedures that are valuable when inferences have to made in the face of uncertainty about the shape and bias of the probability distribution from which the samples have been taken. My argument in this paper is that there is not as much uncertainty about this as has been previously argued. Provided that the sampling distribution is identified approximately correctly, parametric procedures should be more powerful than most robust procedures.

Robust estimates of the central location and confidence interval behave like the standard estimates when the data actually are normally distributed but they are insensitive to the presence of aberrant observations, and they are thus now an accepted part of statistics (Wilcox, 2004). However, their use is not without potential pitfalls. For example, the trimmed mean is very robust to variation in the shape of the frequency distribution and is thus likely to produce a reasonable estimate of the true mean, but the standard deviation (and thus the confidence interval) will be underestimated, sometimes quite dramatically. If this approach is to be adopted, then it may actually be better to use the median-related bisquare biweight (or Tukey biweight) and its associated biweight midvariance (e.g. Hubbell et al. 2002).

A similar caveat applies to the mode, for which there is no analytical estimator of the standard deviation (it should *not* be combined with the usual estimate, as done by Heckman et al. 2001). Both the trimmed mean and mode are amenable to bootstrapping, however, as used for the mode by Hedges et al. (2004), and this is likely to be the preferred method for calculating confidence intervals. Unfortunately, the mode may produce a serious under-estimate of the mean divergence time if the frequency distribution is consistently right-skewed, as shown in Figures 1, 2, 3, 5 and 6. For a lognormal distribution, the mode is in fact further from the geometric mean than is the arithmetic mean. Alternatively, for a lognormal distribution the median should actually be a good estimate of the geometric mean (and vice versa), becoming more so with increasing sample size.

Explicit deletion of outliers is also problematic, although Shaul and Graur (2002: 60) inappropriately present this as being “more rigorous” than the use of a trimmed mean. For example, outlier tests such as Grubb’s test are based on detecting deviations from a normal distribution (Barnett and Lewis, 1994), which is clearly inappropriate if the probability distribution is lognormal—what appears to be an extremely large value, for example, may not necessarily reflect an outlier (although an extremely small value will). Deletion of values can therefore lead to larger problems than the ones that the procedure is intended to solve. As a specific example, Shaul and Graur (2002) provide further re-analysis of the multi-gene data of Wang et al. (1999). In order to assess whether the bird—mammal divergence time estimated from the 29 genes is consistent with the predicted value of 310 MYA, these authors deleted the smallest seven estimates as being inconsistent based on the primate—rodent divergence time and then deleted the largest value because it failed Grubb’s test. They thus produced (from the remaining 21 genes) an estimate of 393 MYA with a 95% confidence interval of 315–471 MYA, which excludes the predicted value. They thus called into serious question the use of secondary calibration points.

However, deleting the seven smallest values *must* produce an upward-biased estimate of the mean. An alternative approach, as I have advocated here, is to take the original multi-gene data at face value and thus to view them as showing stochastic (multiplicative) variation around the geometric mean. A better approach would then be to use all of the data (i.e. 29 genes) and to calculate the geometric mean and confidence interval. This produces a confidence interval of almost exactly the same size as that of Shaul and Graur (2002) but which clearly supports the predicted value (Table 6). Moreover, the reciprocal estimates for the primate—rodent divergence time (Table 6) also well support the predicted time of 110 MYA. Once again, appropriate methodology leads to the opposite conclusion to that from inappropriate methodology, thus emphasizing the practical importance of the issues discussed here.

Finally, it is worth noting that there are other methods for calculating confidence intervals that are relatively independent of the shape of the probability distribution. For example, if the distribution is unimodal then quantifying the curvature of the likelihood surface is a well-known method for estimating confidence intervals when using maximum-likelihood methods of analysis. This profile-likelihood approach is taken by Wall (2003), who emphasizes that it is only approximate.

## Conclusions

Rodríguez-Trelles et al. (2002) have identified an important point about the appropriate scale to be used when estimating divergence (or coalescence) times, but they seem to have over-stated the case when they described it as “a fundamental flaw in the molecular approach to dating” (p. 8112). Here, I have tried to show that the issues addressed by Steel et al. (1996), Haubold and Wiehe (2001), Nei et al. (2001) and Rodríguez-Trelles et al. (2002) are all manifestations of the same underlying cause. That is, these authors offer different perspectives on the same issue, which is that divergence times in phylogenetic studies are lognormally distributed.

For estimates based on a single locus, use of an expected value (i.e. an arithmetic mean) will overestimate the true divergence time and will under-estimate the confidence interval, and such values should always be converted to a lognormal scale (i.e. a geometric mean and its associated confidence interval). This is particularly important if the standard deviation of the estimate is large relative to the mean. However, in practice, any over-estimation of the time is unlikely to be greater than 15% or so, and will usually be much less. Even this can, unfortunately, produce misleading biological conclusions.

For divergence estimates based on multiple loci, the situation is less clear, due to competing sources of variation, but it can be expected that all estimation procedures will converge to the correct solution as the number of loci increases (i.e. the arithmetic and geometric means will converge). Unfortunately, the rate of convergence cannot be predicted, and it seems best in the meantime to use methods that explicitly combine the multiple loci as part of a single estimation procedure, and then to use the geometric mean of the result. The use of robust estimators of central location seems to be a weaker alternative strategy.

Having now identified the most appropriate way to summarize estimates of ancestral divergence times, it becomes important to assess the influence of the other factors that are known to affect the estimation, such as taxon sampling, fossil calibration and among-lineage rate variation. These latter factors affect accuracy rather than precision, and as a result of the work presented here it is now feasible to study their effects in the absence of artefactual over-estimates of divergence time.

## Figures and Tables

**Table 1:** Comparison of methods for estimating confidence intervals on divergence times. Data are shown for six divergence times (millions of years ago, MYA) for pairs of species of the *Arabidopsis* species group (Brassicaceae) based on chalcone synthase sequences, with 95% confidence intervals estimated via bootstrapping. These are compared to confidence intervals estimated using the gamma-based method of Haubold and Wiehe (2001) and the lognormal approximation. Also shown is the geometric mean as calculated from the original estimate and its standard deviation.

Divergence time	Estimate (MYA)^a^	Confidence interval (95%)^a^	Gamma confidence interval^a^	Lognormal confidence interval	Geometric mean (MYA)	Difference (%)^b^
A.t/A.h	5.2	3.2–8.0	3.3–8.0	3.1–7.8	5.0	−4.5
A.t./C.r.	11.3	7.6–16.4	7.5–16.4	7.2–16.2	10.8	−4.7
A.t./A.b.	22.4	15.4–32.2	15.2–31.9	14.6–31.4	21.4	−4.5
C.a./B.v.	6.2	4.2–8.7	4.2–8.8	4.2–8.9	6.1	−1.8
A.t./B.v.	15.0	10.4–21.3	10.1–21.6	9.9–20.8	14.3	−4.4
A.b./B.v.	22.8	15.9–32.5	15.6–32.6	15.1–31.8	21.9	−4.0

a Original data from Haubold and Wiehe (2001).

b Difference between the geometric mean and arithmetic mean, as a percentage of the arithmetic mean.

**Table 2:** Results of best-fit normal and lognormal probability distributions to the frequency histograms obtained by bootstrapping of the angiosperm *rbcL* sequence data reported by Sanderson and Doyle (2001). Three bootstrap analyses were performed, and each was performed separately for the 1st ± 2nd and 3rd codon positions, yielding six frequency histograms, to which both normal and lognormal probability histograms were fitted using maximum likelihood.

Data set	Skewness	Log-likelihood	
		Normal	Lognormal
*Topological uncertainty*			
Positions 1 + 2	1.053	−474.822	−469.264
Positions 3	1.017	−522.394	−509.138
*Character sampling*			
Positions 1 + 2	0.663	−451.489	−448.952
Positions 3	−0.328	−371.652	−374.822
*Taxon sampling and lineage variation*			
Positions 1 + 2	−0.207	−423.770	−425.003
Positions 3	0.404	−418.676	−416.882

**Table 3:** Summary of the results of simulated data for 20 genes, all estimating the same divergence time, based on either the arithmetic or geometric means for each gene. Results are shown for the maximum-likelihood fitting of the normal and lognormal probability distributions to the frequency histogram of the 20 time estimates. Also shown are the summary statistics of the 20 estimates; the time scale is relative (i.e. unitless until a substitution rate is specified for each gene) with a target time in the simulations of 10 units. These are all compared to the distance-based estimator described by Nei et al. (2001:2498).

Summary	Arithmetic means	Geometric means	Distance estimator
*Log-likelihood*			
Normal	−51.89	−19.97	−0.44
Lognormal	−46.09	−18.99	−0.49
*Mean divergence time*			
Arithmetic	11.5	10.3	10.1
Geometric	11.2	10.3	10.1
*Standard deviation*			
Arithmetic	3.3	0.7	0.3

**Table 4:** Results of analyses based on the multiple-gene data set for various higher taxa reported by Wang et al. (1999). For each of the six pairwise comparisons, the arithmetic and geometric averages are shown, based on arithmetic node-age estimates for each gene (millions of years ago, MYA), along with the results of the maximum-likelihood fitting of the normal and lognormal probability distributions to the frequency histogram of the time estimates.

Divergence time	No. genes	Skewness	Log-likelihood		Mean (MYA)		Difference (%)^a^
			Normal	Lognormal	Arithmetic	Geometric	
Chordate—Arthropod	50	3.687	−402.1	−382.8	1099	945	−14.1
Chordate—Nematode	25	0.523	−188.9	−190.6	1154	1049	−9.1
Arthropod—Nematode	18	0.833	−137.0	−136.4	1252	1157	−7.6
Metazoa—Fungi	55	3.349	−456.5	−444.2	1631	1419	−13.0
Metazoa—Plant	49	2.333	−410.9	−399.6	1680	1425	−15.2
Fungi—Plant	38	0.627	−306.8	−310.2	1663	1450	−12.8

a Difference between the geometric mean and arithmetic mean, as a percentage of the arithmetic mean.

**Table 5:** Estimated divergence times (millions of years ago) and 95% confidence intervals for the primate—rodent divergence (expected to be 110 MYA), based on either the arithmetic or geometric means for each of the 29 genes and using either the arithmetic or geometric average across the genes. Also shown is the concatenated multigene estimate. The original data are from Glazko and Nei (2003).

Method	Arithmetic means	Geometric Means
Arithmetic average	128.4 (113.7–143.1)	125.2 (110.4–138.0)
Geometric average	122.7 (108.8–138.3)	119.9 (106.7–134.8)
Multigene estimate	117.7 (109.6–125.8)	117.6 (109.8–126.0)

**Table 6:** Estimates and statistical significance of two divergence times (millions of years ago) for 29 genes, based on the analyses of Shaul and Graur (2002) but with the addition of confidence intervals. T1 is the primate—rodent divergence and T2 is the bird—mammal divergence. The original data are from Wang et al. (1999).

Locus name	Geometric mean and 95% confidence interval		Statistical significance^a^	
	T1 (MYA)	T2 (MYA)	T2 > T1	T1 > T2
Aldehyde dehydrogenase	143–215–325	100–151–229		
Aldolase	28–62–138	209–467–1043	*	
Alkaline phosphatase	71–103–150	219–318–462	*	
Alpha actinin	138–261–495	62–117–222		
Amidophosphoribosyl transferase	66–104–164	197–310–490	*	
Aminolevulinate synthetase	146–204–286	116–162–227		
Aspartate aminotransferase	85–133–209	155–243–381		
Dihydrofolate reductase	62–112–202	154–279–505		
Disulfide isomerase	71–112–175	185–289–453	*	
DNA polymerase gamma	92–131–185	183–263–378		
Enolase	114–213–398	77–145–270		
Ferritin heavy chain	65–162–405	68–169–423		
Fructose-2,6-bisphosphatase	39–63–105	305–503–830	*	
Furin	55–80–116	284–411–596	*	
Glutamate dehydrogenase	16–38–92	319–739–1712	*	
Glutamine synthetase	105–181–312	101–175–301		
Glyceraldehyde-3-phosphate dehydrogenase	107–214–430	70–140–282		
Lactate dehydrogenase	63–114–206	151–273–494		
Na-K ATPase alpha chain	60–118–235	129–255–505		
Na-K ATPase beta chain	7–14–27	1106–2190–4337	*	
P53	72–102–145	228–324–461	*	
P65	39–52–72	463–639–881	*	
Phosphoenolpyruvate carboxykinase	114–166–243	135–197–288		
Phosphoglycerate kinase	26–53–109	283–567–1135	*	
Pyruvate kinase	40–69–119	264–458–794	*	
Transcription factor Eryf1	32–51–79	408–639–1001	*	
Transglutaminase	89–113–144	234–297–378	*	
Triosephosphate isomerase	59–124–258	117–247–524		
Tryptophan hydroxylase	118–182–282	115–178–275		
Geometric average and confidence interval	82–104–133	238–302–384		
minus outlier (Na-K ATPase beta chain)	92–112–137	231–282–343		

a Statistical significance is based on whether the lognormal 95% confidence intervals for the two times overlap (=not significant, left blank) or not (=significant, shown with an asterisk).
